# Correction: Efficient removal of cobalt from aqueous solution using β-cyclodextrin modified graphene oxide

**DOI:** 10.1039/c9ra90091h

**Published:** 2019-12-12

**Authors:** Wencheng Song, Jun Hu, Ying Zhao, Dadong Shao, Jiaxing Li

**Affiliations:** School of Nuclear Science and Technology, University Science and Technology of China 230026 Hefei P. R. China; Key Laboratory of Novel Thin Film Solar Cells, Institute of Plasma Physics, Chinese Academy of Sciences P.O. Box 1126 230031 Hefei P. R. China shaodadong@126.com +86-551-5591310 +86-551-5592788

## Abstract

Correction for ‘Efficient removal of cobalt from aqueous solution using β-cyclodextrin modified graphene oxide’ by Wencheng Song *et al.*, *RSC Adv.*, 2013, **3**, 9514–9521.

The authors regret that Fig. 1 and 3 were incorrect in the original article. The SEM images of both GO and β-CD, and the Raman spectra of both, were confused with other samples. The correct versions of Fig. 1 and 3 are presented below.

**Fig. 1:**
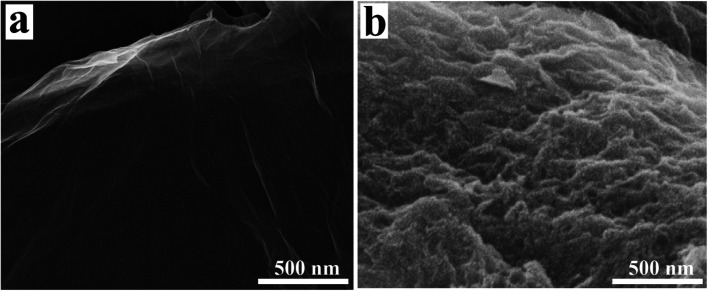
SEM images of (a) GO and (b) β-CD-GO.

**Fig. 2:**
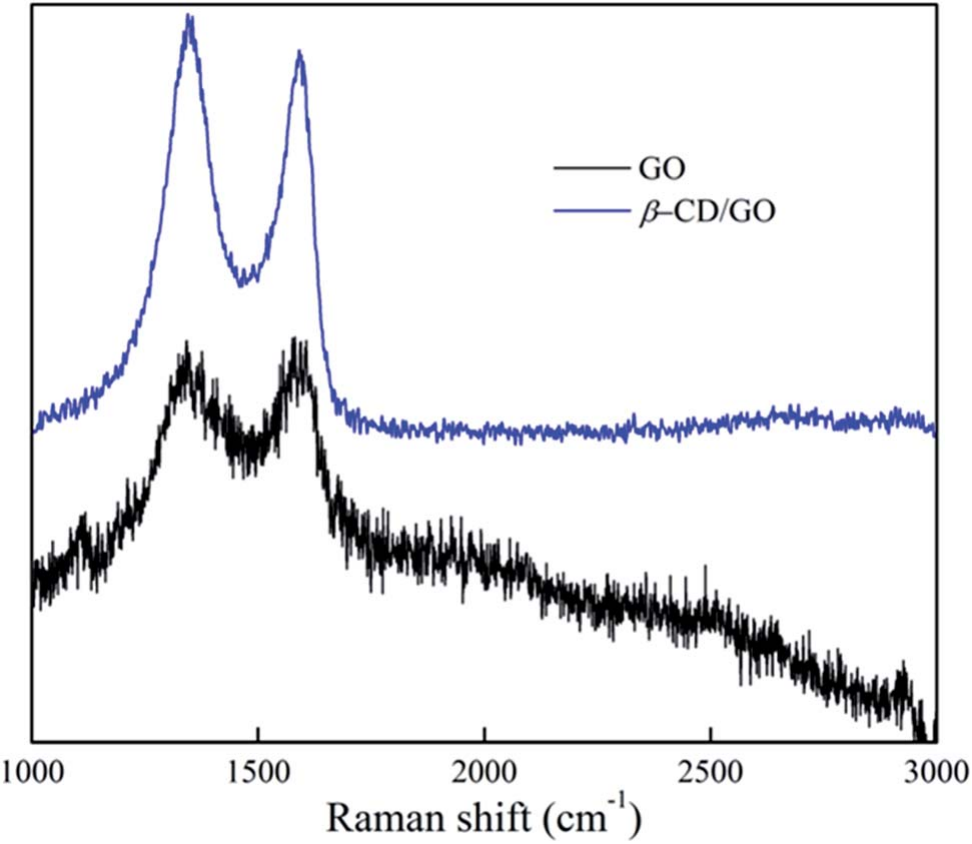
Raman spectra of GO and β-CD-GO.

The Royal Society of Chemistry apologises for these errors and any consequent inconvenience to authors and readers.

## Supplementary Material

